# Internet-based behavioural activation to improve depressive symptoms and prevent child abuse in postnatal women (SmartMama): a protocol for a pragmatic randomized controlled trial

**DOI:** 10.1186/s12884-021-03767-9

**Published:** 2021-04-20

**Authors:** Erika Obikane, Toshiaki Baba, Tomohiro Shinozaki, Soichiro Obata, Sayuri Nakanishi, Chie Murata, Emiko Ushio, Yukio Suzuki, Norihito Shirakawa, Mari Honda, Natsu Sasaki, Daisuke Nishi, Heather O’Mahen, Norito Kawakami

**Affiliations:** 1 Department of Mental Health, Graduate School of Medicine, The University of Tokyo, Tokyo, Japan; 2 Bureau of International Health Cooperation, National Center for Global Health and Medicine, 1-21-1, Toyama, Shinjuku-ku, Tokyo, 162-8655 Japan; 3 Department of Information and Computer Technology, Faculty of Engineering, Tokyo University of Science, Tokyo, Japan; 4 Perinatal Center for Maternity and Neonates, Yokohama City University Medical Center, Yokohama, Japan; 5 Department of Obstetrics and Gynecology, Yokohama Municipal Citizen’s Hospital, Yokohama, Japan; 6 Department of Obstetrics and Gynecology, Yokohama City University Graduate School of Medicine, Yokohama, Japan; 7 Yokohama City Mental Health and Welfare Centre, Yokohama, Japan; 8 Mood Disorders Centre, University of Exeter, Exeter, UK

**Keywords:** Behavioural activation, Cognitive behavioural therapy, Postnatal depression, Child abuse, Implementation, Internet-based psychotherapy

## Abstract

**Background:**

Child abuse and postnatal depression are two public health problems that often co-occur, with rates of childhood maltreatment highest during the first year of life. Internet-based behavioural activation (iBA) therapy has demonstrated its efficacy for improving postnatal depression. No study has examined whether the iBA program is also effective at preventing child abuse. This study aims to investigate whether iBA improves depressive symptoms among mothers and prevents abusive behaviours towards children in postpartum mothers in a randomized controlled trial, stratifying on depressive mood status. The study also evaluates the implementation aspects of the program, including how users, medical providers, and managers perceive the program in terms of acceptability, appropriateness, feasibility, and harm done.

**Methods:**

The study is a non-blinded, stratified randomized controlled trial. Based on cut-off scores validated on Japanese mothers, participants will be stratified to either a low Edinburgh Postnatal Depression Scale (EPDS) group, (EPDS 0–8 points) or a high EPDS group (EPDS ≥9 points). A total of 390 postnatal women, 20 years or older, who have given birth within 10 weeks and have regular internet-access will be recruited at two hospitals. Participants will be randomly assigned to either treatment, with treatment as usual (TAU) or through intervention groups. The TAU group receives 12 weekly iBA sessions with online assignments and feedback from trained therapists. Co-primary outcomes are maternal depressive symptoms (EPDS) and psychological aggression toward children (Conflict Tactic Scale 1) at the 24-week follow-up survey. Secondary outcomes include maternal depressive symptoms, parental stress, bonding relationship, quality of life, maternal health care use, and paediatric outcomes such as physical development, preventive care attendance, and health care use. The study will also investigate the implementation outcomes of the program.

**Discussion:**

The study investigates the effectiveness of the iBA program for maternal depressive symptoms and psychological aggression toward children, as well as implementation outcomes, in a randomized-controlled trial. The iBA may be a potential strategy for improving maternal postnatal depression and preventing child abuse.

**Trial registration:**

The study protocol (issue date: 2019-Mar-01, original version 2019005NI-00) was registered at the UMIN Clinical Trial Registry (UMIN-CTR: ID UMIN 000036864).

## Background

Child abuse is a challenging issue in public health as it affects the physical, psychological, and social health outcomes of individuals for a lifetime [1–9]. From a societal perspective, child abuse imposes a substantial financial burden on societies as it creates high direct and indirect costs in low, middle, and high-income countries [10–12]. Existing research has identified the following characteristics as being associated with a higher risk for child abuse: parental depression, anxiety and anger-hyperactivity, parent stress, poor parent–child interactions, and low social support [13]. However, there is presently no solution for how to prevent child abuse right after birth.

Postnatal depression is another critical problem closely related to child abuse [14, 15]. Depression is the leading cause of disease-related disabilities among women [16, 17]. Previous studies indicated that women with postpartum depression were at higher risk for lower physical and psychological health, substance abuse, and suicidal behaviours [18–22]. Child abuse and postnatal depression often co-occur, and the rate of childhood maltreatment is highest during the first year of life [23, 24]. Children born to depressed mothers were at higher risk for prematurity, delayed physical, motor, and cognitive development [22]. Moreover, postnatal depression among mothers was associated with a significantly increased risk for harsh parenting, physical and psychological abuse [14, 15, 25–27].

Previous evidence has demonstrated that cognitive behavioural therapy (CBT) is effective for improving depressive symptoms among postnatal women [28–33], and emerging evidence has shown that it also reduces maternal stress [34, 35]. Behavioural activation (BA) is a simple but effective psychotherapy that follows the basic CBT principles but does not include cognitive restructuring. The practical principles of BA may be of particular use for postnatal women, who face multiple competing demands on their time and attention. Further, the parsimonious, simple nature of BA supports its straightforward application with a wide range of mental health providers, contributing to its promise as a highly scalable intervention [13]. Moreover, as BA facilitates participants to engage in positive behaviours and works on problem-solving strategies when barriers to implementation of these behaviours are encountered, it may increase positive parent–child interactions, possibly enhancing the bonding relationship between mothers and children and improving child outcomes. In a large study of BA in postnatally depressed mothers who were also randomly assigned to receive either relaxation or a video-feedback parent–child intervention, both conditions resulted in improved cognitive, behavioural and attachment outcomes for the child at 2 years of age [36]. These results suggest that BA may help to support the parent–child relationship underpinning attachment, possibly preventing parental abusive behaviours toward their children.

Internet-based psychotherapy has become an alternative approach to face-to-face psychotherapy or other interventions due to its strengths in accessibility, reach, and cost effectiveness [28, 32, 37–41]. These approaches have gained widespread application during the COVID-19 pandemic. Users can choose their device, time, and place to receive the program depending on their needs. Providers of the program, on the other hand, can deliver the application to the mass target population with fewer human resources and lower cost. A number of studies have now demonstrated that internet-based cognitive behavioural therapy (iCBT) [38, 42], which contains internet-based behavioural activation (iBA) is an effective treatment for prenatal depression, and that iBA itself is an effective treatment for postnatal depression [31, 32].

Internet-based psychotherapy not only reduces practical barriers to access for users but may also reduce psychological barriers. Previous evidence has shown that many women with postnatal depression struggle with stigmatization, and this can be an obstacle for health care providers attempting to connect depressed mothers with medical care [43, 44]. In qualitative studies of iBA for postnatal depression, mothers who reported struggling with stigma stated that they found internet intervention more acceptable than face-to-face intervention [45]. We expect that internet-based interventions including smartphones, which have become such standard communication tools, would make it easier for mothers in need to seek care for their mental health. Internet-based psychotherapy, however, has its own challenges. Many studies have described the high attrition rate of internet-based psychotherapy, ranging from 9.7 to 78.9% of the participants, as a weakness of web-based interventions [40, 46]. Internet-based interventions demonstrated improved retention rates with human support [28, 32, 41, 47–49]; however, the adequate intensity of support needed to achieve the goal is still unknown. As the implementation of the program involves not only the users but also medical providers and hospital managers, it is essential to understand how the program is perceived in terms of acceptability, appropriateness, feasibility, and potential harm perceived by these stakeholders.

## Methods/design

### Objectives

The study is a 6-month follow-up stratified randomized controlled trial at multiple medical centres. A web-based behavioural activation program for smartphones with therapist support will be developed for postnatal mothers in Japan, based on a previous study [32]. The aims of the study are as follows:

1. To investigate the effect of an internet-based behavioural activation program on improving depressive symptoms and preventing abusive behaviours toward their children as co-primary outcomes, as well as parental stress, bonding relationship, health-related quality of life (QOL), and child developmental measures as secondary outcomes.
2. To investigate how the medical and social characteristics of participants influence implementation aspects (acceptability, appropriateness, and feasibility). We have set two co-primary outcomes in this trial, as the effects on both mothers and children are equally important. The hypotheses of the study are as follows:

(H1) The web-based behavioural activation program for smartphones will significantly improve depressive symptoms 24 weeks after the initiation of the program among postnatal mothers with EPDS scores ≥9 points in the intervention group as compared to those in the TAU group; and.

(H2) The smartphone-based behavioural activation program will significantly prevent abusive behaviours toward children at 24 weeks after the initiation of the program among all the postnatal mothers in the intervention group as compared to those in the TAU group.

### Study design

The study is a non-blinded, parallel group, stratified randomized controlled trial. Based on the most recent score of the Edinburgh Postnatal Depression Scale (EPDS) at recruitment, participants will be stratified to either low EPDS group (EPDS 0–8 points) or high EPDS group (EPDS ≥9 points) after completing the baseline survey. The threshold of 8/9 points was determined fit according to the previous validation studies conducted in Japan [50, 51].

The allocation ratio of the intervention group to the control group is set as 1:1. Participants will be asked to answer the online follow-up surveys at 12 and 24 weeks after the initiation of the program. The protocol of the study was registered at UMIN Clinical Trial Registry (UMIN-CTR: ID UMIN 000036864). As of February 2021, we are in the process of recruiting participants. The study protocol follows the Standard Protocol Items for Randomized Trials (SPIRIT) guideline checklists.

### Study setting

The study will be conducted in the department of Obstetrics and Gynecology at two tertiary care hospitals that provide maternal and foetal intensive care in Yokohama, Japan.

### Inclusion criteria

- Age ≥ 20 years old at recruitment (Age of adulthood is 20 years or older in Japan).
- Postnatal women who had given birth within 10 weeks of recruitment.
- Have regular internet-access via smartphones or other internet devices.
- Currently living with the new-born baby.
- Fluent in Japanese (Able to understand the content and work on the program).

### Exclusion criteria

- Current suicidal intent at recruitment.
- Currently not living with the new-born baby for any reason, including if the baby is still receiving care in the neonatal intensive care unit.
- Currently receiving public livelihood assistance or public assistance for delivery cost.

### Procedure

**Fig. 1:**
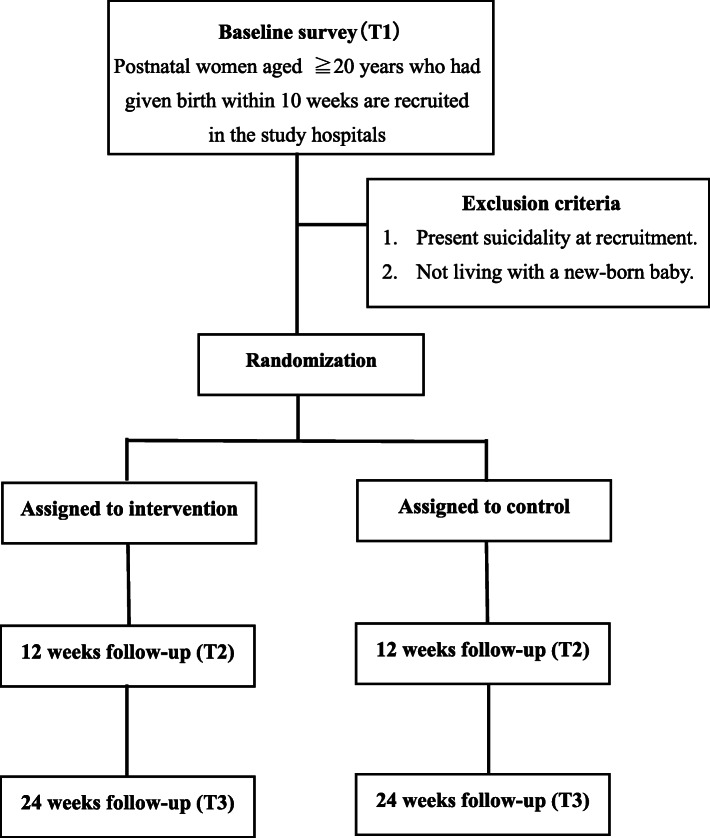
Flow chart of participants

The flow chart of participants is presented in Fig. 1. Women who have given birth and arrive at the study sites for postnatal visits (usually at 4 weeks after delivery), and fulfilled the eligibility criteria, will be considered as possible candidates for recruitment. Potential candidates will receive handouts with brief information on our research study from their health care providers (medical doctor, nurse, midwife, or medical assistants). If they are interested in participating in the study, they will be given access to a Quick Respond code. This machine-readable optical barcode can connect to our information site on the internet and register their e-mail addresses for further information. If candidates agree to participate after reading the full information on our study, they will go through the online informed-consent process by confirming that they have read and understood the statements and clicking the “agree” button. Consenting participants will be asked to answer a baseline online survey (T1). Participants will then be allocated to either an intervention group or a treatment as usual (TAU) control group, stratified according to EPDS scores at recruitment (low EPDS: 0–8 points, high EPDS ≥9 points), and hospital codes. To ensure allocation concealment, research assistants will register group codes into the program using the block randomization sheet created by a biostatistician.

This study also conducts a short survey on the implementation of the program with medical providers and hospital managers of the study during the recruitment period. The providers and hospital managers will be asked to browse the content of the program with their own smartphones and answer the short survey consisting of implementation and usability scale outcomes.

### Intervention program

The intervention program (SmartMama) is developed based on the UK behavioural activation program for postnatal depression (Client manual: Self-help booklet for postnatal depression by Heather O’Mahen [52]), adjusted for Japanese postpartum mothers who use smartphones [30]. The program consists of 12 weekly online sessions (each session requires 30 min) with online assignments and feedback from trained therapists during the course.

The contents of the program are shown in Table 1. In Sessions 1 and 2, participants learn about basic concepts of behavioural activation—that mood, feeling, and behaviours are closely related. They practice how to track their mood, feelings, and actions using a mood diary. In Sessions 3 and 4, participants learn about Triggers–Reactions–Avoidance Patterns (TRAPs) model, how the trigger event leads to a certain mood, often causing avoidance behaviours among participants. Participants break down the patterns of the situations in which they felt stuck, apply the TRAP model to explain their cases, and understand that their actions and mood are closely linked. In Sessions 5 and 6, participants learn to develop alternative coping strategies to handle the situations and shift from avoidance patterns to alternative coping. In Sessions 7 and 8, participants learn about strategies for effective communication and to ask for support. In Sessions 9 and 10, participants identify their goals of being good-enough mothers and turn their avoidance behaviours into alternative coping. In Sessions 11 and 12, participants learn about strategies to stay well, recognize their warning signals, and prepare for the future.

**Table 1:** The contents of the behavioral activation therapy program

Sessions	Title	Contents
0	Introduction	Introduction on changes that occur in perinatal periods that affect your activities
1	Understanding the depression cycle	Learning about relationship between your mood and behaviours, and exercise to fill out the mood diary.
2	Self-monitoring, self-care	Learning about self-monitoring skills.
3	Identifying the patterns to get stuck in you depressive mood	Understanding the Triggers-Reactions-Avoidance Patterns (TRAPs) model.
4	Case study	Case studies on how a trigger event induces feeling (reaction), causing avoidance pattern. Writing about your own case using a TRAP model.
5	Altenative coping stratigies	Looking for althernative activities as a mother
6	Case Study	Case studies on using alternative coping. Analyse your own case to come up with althernative behaviours.
7	Support and communication	Understanding your need for support and how communication needs to be changed during perinatal period.
8	Communication strategies	Learning communication strategies to ask for help and support in a comfortable way.
9	Being a “good-enough” mom	Identifying your own “mummy goals” and using althernative coping strategies to meet them.
10	TRAPs aroug being a good enough mom	Looking at your TRAP case and how to turn into altenative coping behaviours.
11	Staying well	Looking back over the course and identify the strategies that were effective and helpful.
12	Planning ahead	Identify your warning signals and plan your strategies to feel better.

### Intervention group

Participants in the intervention group will be asked to work on 12 weekly sessions of the online application-based behavioural activation programs, as described in the previous section. Each week, participants in the intervention group will receive e-mail reminders that a new session has become available. Further, they will be asked to answer short screening questionnaires on their depressive symptoms each week. If the participants present with severe depressive symptoms and suicidal thoughts, alert messages and information on mental health care will appear on the screen, as well as automatically notifying investigators of the information. If participants have questions about the program, they can ask the therapists within the program. They will receive answers within 1–2 days. Participants are allowed to receive treatment as usual, including inpatient or outpatient consultation, medication, or psychotherapy during the study period.

### Control (TAU) group

Participants in the TAU group receive weekly messages and will be asked to answer short screening questionnaires on their depressive symptoms each week. Similar to the intervention group, alert messages and information on mental health care will be shown if the participants present with severe depressive symptoms or suicidal thoughts. They are also allowed to receive treatment as usual, including inpatient and outpatient medical care.

### Outcomes

The schedule of assessments is summarized in Table 2. The co-primary outcomes are measured in the baseline, with 12- and 24-week follow-up online surveys. Participants who have not completed follow-up surveys will receive weekly reminders to complete their surveys.

**Table 2:** Schedule of enrolment, interventions, and assessments

		Enrolment	Allocation	Post-allocation	
TIMEPOINT		T0	0	12 weeks follow-up (T1)	24 weeks follow-up (T2)
**ENROLMENT:**					
Eligibility screen		X			
Informed consent		X			
Allocation			X		
**INTERVENTIONS:**					
Intervention group					
Control group					
**ASSESSMENTS:**	**Aim**				
**Co-primary outcomes**					
EPDS	Maternal depressive symptoms	X		X	X
CTS-1	Psychological aggression toward children	X		X	X
**Secondary outcomes**					
**Maternal**					
PHQ-9	Depresssive symptoms	X		X	X
PSI-SF	Parent stress	X		X	X
MIBS-J	Mother-to-infant bonding	X		X	X
EQ-5D-5L	Quality of life	X		X	X
Use of inpatient and outpatient medical care in the past 3 months	Medical cost	X		X	X
**Paediatric**					
Height, body weight	Physical development	X		X	X
Type of nutrition (breast milk, formula, both, baby food)	Nutritional status	X		X	X
Attendance of well-baby visits at 1, 3, 6–7, 9–10 months)	Health check-up status	X		X	X
Immunization record (Hib, pneumococcus, DPT-IPV, rotavirus, BCG)	Immunization status	X		X	X
Use of inpatient and outpatient medical care for injuries, foreign body ingestion, burn, and drowning in the 3 months	Medical cost for injuries	X		X	X
**Implementation**					
Implementation outcome scale for Digital Mental Health (IDMH) for users	Acceptability, appropriateness, feasibility, overall satisfaction, and harms			X	

*EPDS* Edinburgh Postnatal Depression Scale, *CTS-1* Conflict Tactics Scale, *PHQ-9* Patient Heatlh Questionnaire-9, *PSI-SF* Parent stress index-short form Japanese version, *MIBS-J* Mother-to-Infant Bonding Scale Japanese version, *EQ-5D-5L* EuroQol-5 dimension-5 level

### Co-primary outcomes

The co-primary outcomes of the study are the depressive symptoms at 24 weeks after initiation of the program and aggression toward their children at 24 weeks after the initiation of the program. We have set two co-primary outcomes, as both maternal and child outcomes were considered equally important.

### Depressive symptoms

The depressive symptoms of participants will be assessed with the EPDS score measured in online surveys. The EPDS is a reliable and valid screening tool that is widely used in perinatal medical and healthcare settings in Japan [50, 53]. Participants are asked to self-report their depressive symptoms on a scale of 0 to 3 for 10 items (total score range being 0–30).

### Psychological aggression toward children

The study uses psychological aggression toward children as outcomes of abusive behaviours toward children. Psychological aggression toward children will be measured by the psychological aggression item of the Conflict Tactic Scale I (CTS-1), a Japanese version developed by Kitamura, et al. [54]. The CTS-1 is a commonly used self-report measure for abusive behaviours toward children that has been tested for its validity [55, 56]. The participants will be asked to answer the seven items on psychological aggression, on a scale of 0 to 6, with a higher score indicating more severe psychological aggression.

### Secondary outcomes

#### Maternal depressive symptoms

Depressive symptoms of participants will be evaluated by the Patient Health Questionnaire-9 (PHQ-9). The PHQ-9 is a brief and straightforward self-report instrument to screen and measure the severity of depressive symptoms. The PHQ-9 correlates with the Diagnostic and Statistical Manual of Mental Disorders, Fourth Edition (DSM-IV) criteria for the major depressive disorder [57, 58].

#### Parental stress

Parental stress will be assessed by the Japanese version of the Parental stress index-short form (PSI-SF). The PSI-SF is a self-report screening tool that consists of 19 questions— 9 questions for child characteristics, and 10 questions for parent characteristics on a 5-point Likert scale. A previous study indicates acceptable reliability and internal validity [59].

#### Bonding relationship

Mother-to-infant bonding relationship will be measured by the Mother-to-Infant Bonding Scale Japanese version (MIBS-J). The MIBS-J is a self-report questionnaire that consists of 10 questions to assess a mother’s feelings toward her child on a 4-point Likert scale. The MIBS-J is widely used in health care settings in Japan, and has acceptable reliability and validity [60].

#### Physical assault

Maltreatment measures including both minor and major physical assault are measured with 9 physical assault items of CTS-1 [54–56], as mentioned in the psychological aggression section. The participants will be asked to answer the nine items on physical assault on a scale of 0 to 6, with a higher score indicating more severe physical assault.

#### Quality of life

The quality of life is measured with the EuroQol-5 dimension-5 level (EQ-5D-5L). The EQ-5D-5L is an instrument consisting of five items with five levels to evaluate the health status of the population. The five dimensions include mobility, self-care, usual activities, pain/discomfort, and anxiety/depression. It is widely used and has acceptable reliability and validity according to previous studies [61, 62].

#### Healthcare use for mothers

Healthcare use for mothers will be evaluated by questions on the inpatient and outpatient healthcare use for psychiatric problems faced by the participants (the number of days for outpatient visits and hospitalization), or home visitation by public health nurses in the past 3 months during the 12–24 week surveys.

#### Paediatric physical development

The physical development of children will be assessed by standard deviation scores for body weight and height based on the recent records of children’s body weight and height during the 12-week and 24-week follow-up surveys.

#### Health check-up attendance

Health check-up attendance will be evaluated by asking questions about whether children have attended well-baby check-ups at 3, 6, and 9 months (if applicable) during the 12-week and 24-week follow-up surveys.

#### Immunization status

Immunization status will be evaluated by asking questions about whether children have completed immunization for Hemophilus influenzae type b, *Streptococcus pneumoniae*, diphtheria, tetanus, pertussis, and polio (DPT-IPV) during the 12–24 week follow-up surveys.

#### Healthcare use for children

The healthcare use of children will be evaluated by asking questions about the inpatient and outpatient healthcare use for injuries, fractures, burns, foreign body ingestions, skin problems, or nutrition problems in the past 3 months. Healthcare questions will be administered during the 12–24 weeks of follow-up surveys.

#### Implementation outcomes

Implementation outcomes will be measured by the Implementation Outcome Scale for Digital Mental Health (iOSDMH) and the System Usability Scale (SUS). The iOSDMH has been developed to cover the key concepts of implementation in digital mental health, including acceptability, appropriateness, and feasibility, based on a previous systematic review [63]. The iOSDMH evaluates these implementation concepts among different levels of stakeholders involved in the intervention process, including users, providers, and managers. Participants will be asked to answer the questionnaires consisting of 19 items from the iOSDMH for users on acceptability, appropriateness, feasibility, and harms, as well as 10 items on usability at the 12-week follow-up survey. Medical providers will be asked to answer the iOSDMH questionnaires that have been specifically developed for providers’ perceptions on appropriateness, feasibility, and harm of the program, as well as SUS items. Hospital managers will be asked to answer the iOSDMH questionnaires for managers that have been developed to evaluate the perceptions of managers, as well as SUS items.

#### Sample size calculation

As the study has two co-primary outcomes, we calculated each sample size needed to improve the depressive symptoms of mothers and to prevent abusive behaviours toward children based on the effect size from a previous study.

The study reported the effect size of the internet-based behavioural activation for postnatal depression as d = 0.55 [32]. With a significance level of 0.05, the statistical power of 80%, and a follow-up rate of 85%, the sample size needed to detect efficacy for postnatal depression in the high EPDS group is 128 participants (64 participants for each arm). (Sample size calculation was carried out using the G*Power 3.1.9.2.)

No previous study examined the effect of iBA for preventing child abuse, so we used the effect size d = 0.56 of cognitive behavioural therapy for anger from a randomized controlled trial [64]. We estimated the effect size of the iBA for psychological aggression as d = 0.50 for the high EPDS group based on the previous study [64], and d = 0.40 for the low EPDS group. With a significance level of 0.05, the statistical power of 80%, and a follow-up rate of 80%, the required sample size is 150 participants for the high EPDS group (75 participants per arm) and 240 participants for the low EPDS group (120 participants per arm).

Finally, we expected the sample size needed to detect the change in both depressive symptoms and abusive behaviours would be 150 participants for the high EPDS group and 240 participants for the lower EPDS group.

#### Randomization

We apply a stratified permuted block randomization for this study. Participants are classified into four strata based on the hospital codes (two hospitals) and the EPDS groups (lower EPDS: 0–8 points, the higher EPDS: ≥ 9 points). The biostatistician will create a stratified block random table, and the research assistant will allocate participants into either the intervention or TAU group according to the Table. A list of participants allocated to the intervention and TAU groups will be password-locked in the computer and will be blinded to the researchers.

### Statistical methods

#### Clinical efficacy

For primary outcomes, an analysis of covariance (ANCOVA) will be conducted to compare the mean differences of co-primary outcomes at 24 weeks follow-up between the intervention and TAU groups after adjusting baseline scores. The study sets two co-primary outcomes, including the EPDS scores at 24 weeks after the initiation of the program and psychological aggression toward children measured by the CTS-1 scores at the 24 weeks follow-up survey. The order of statistical testing is as follows: (1) the EPDS scores at 24 weeks follow-up; (2) CTS-1 scores at 24 weeks follow-up. If we find statistical significance for the EPDS scores at the predefined 0.05 level, then we will conduct statistical testing for the CTS-1 scores. If we do not find statistical significance for the EPDS scores, we will conduct the ANCOVA for the CTS-1 score as a complementary analysis. Given the fixed-sequence testing procedure, the study will not adjust for the multiplicity of each statistical test.

For secondary outcomes, the ANCOVA will be conducted to compare secondary outcome measures (Table 2) between the TAU and intervention groups after adjusting baseline scores. Statistical analyses will be performed using the SAS version 9.4 (SAS Institute, Cary, North Carolina, USA).

#### Subgroup analysis

For primary outcomes, we will conduct a subgroup analysis using initial EPDS, socio-economic factors, adverse childhood experiences, and social support.

#### Sensitivity analysis

We will use a complete analysis based upon the assumption missing completely at random. We will conduct a sensitivity analysis using an inverse probability weighted generalized estimating equation to estimate the dropout mechanism. As previous study has indicated that socio-economic status such as education and household income influenced the attrition rate [30] and we supposed that initial depressive symptoms would possibly influence adherence to the program, this study will use baseline scores of the EPDS, and socio-economic status (highest education, income) as weighting factors.

#### Data confidentiality

Information on individuals acquired through this study will be anonymized. The correspondence table of program ID and individual information will be created and stored in the Department of Mental Health, Graduate School, University of Tokyo. The research data collected in online surveys will be temporarily stored in the Amazon Elastic Compute Cloud system (Amazon Web Services, Inc.). The data will be moved to a password-locked PC in the Department of Mental Health, Graduate School, University of Tokyo. Only principal investigators will have access to the final data sets.

#### Data monitoring

This study does not have an external data monitoring board; however, we will have regular meetings to discuss our current status of recruitment and data collection with external medical providers and researchers.

## Discussion

The strength of the study is that it evaluates the effectiveness of the iBA program for maternal depressive symptoms and psychological aggression toward children in a randomized-controlled trial. The study also intends to examine the implementation outcomes of the iBA program, which is essential in delivering the program to the population in need. The limitation of the study is that all the outcomes are measured by the self-report scales. Although all the postnatal mothers are recruited in the postnatal check-up at one month after delivery, the time that they complete the baseline online survey and finish enrolment may differ. This may influence the baseline EPDS scores. Moreover, the participants are to be recruited in relatively large hospitals, and the finding of the study may not be generalizable to the postnatal women having deliveries at smaller hospitals and clinics.

The results of the study may be relevant and beneficial to the health care needs of postnatal women and their children, as well as in providing future directions to health policy for postnatal care and child abuse prevention.

## Data Availability

The manuscript consists of a trial protocol. Data sharing is not applicable; no datasets were generated or analysed in the preparation of this protocol.

## Abbreviations

BA: behavioural activation
CBT: cognitive behavioural therapy
iBA: internet-based behavioural activation
iCBT: internet-based cognitive behavioural therapy
EPDS: Edinburgh Postnatal Depression Scale
TAU: treatment as usual
QOL: quality of life
TRAPs: Triggers-Reactions-Avoidance Patterns
CTS-1: Conflict Tactics Scale 1
PHQ-9: Patient Health Questionnaire-9
PSI-SF: Parental stress index-short form
MIBS-J: Mother-to-Infant Bonding Scale Japanese version
EQ-5D-5L: EuroQol-5 dimension-5 level
DPT-IPV: diphtheria, tetanus, pertussis, and polio
iOSDMH: Implementation outcome scale for Digital Mental Health
SUS: System Usability Scale
ANOVA: analysis of covariance
